# Risk of Erythritol-Associated Ischemic Stroke: Integrated Genetic, Transcriptomic, and Machine Learning Evidence

**DOI:** 10.3390/ijms27177811

**Published:** 2026-08-31

**Authors:** Ao Zhong, Fangyang Yu, Chuyue Xia, Xiang Ma, Qiucheng Zhu, Peilin Du, Ruonan Wang, Si Jin

**Affiliations:** Department of Endocrinology, Institute of Geriatrics, Key Laboratory of Vascular Aging of the Ministry of Education, Liyuan Hospital of Tongji Medical College, Huazhong University of Science and Technology, Wuhan 430077, China

**Keywords:** erythritol, ischemic stroke, inflammation, immune infiltration, multi-omics, machine learning

## Abstract

Erythritol is a widely used low-calorie sugar substitute, but its relationship with cerebrovascular risk remains uncertain. We investigated the association between genetically predicted erythritol levels and ischemic stroke and explored stroke-related molecular features using an integrative bioinformatics framework. Two-sample and multivariable Mendelian randomization were performed across cardiovascular–kidney–metabolic outcomes, followed by target prediction, enrichment and protein–protein interaction analyses, transcriptomic profiling, machine-learning feature selection, SHAP interpretation, immune-cell analysis, gene set variation analysis, and exploratory molecular docking. Genetically predicted erythritol showed the strongest association with stroke among the tested sweetener-related traits (OR = 1.246, 95% CI: 1.101–1.410, *p* < 0.001) and remained significant after adjustment for selected hemodynamic, glycometabolic, and lipid-related traits. Downstream analyses highlighted inflammatory, oxidative-stress, hypoxic, and vascular-injury pathways and prioritized MMP9, TLR4, and HIF1A as a reproducible stroke-related three-gene signature. These downstream bioinformatic findings are exploratory and do not establish erythritol-specific molecular regulation. Overall, the results support an association between genetically predicted erythritol levels and ischemic stroke and identify candidate pathways and genes for further investigation; the MR exposure should not be interpreted as direct evidence that dietary erythritol intake causes stroke.

## 1. Introduction

Stroke is a leading cause of disability and death worldwide, and accumulating evidence indicates that inflammation, immune cell recruitment, oxidative stress, endothelial dysfunction, and hypoxia-related vascular injury are key contributors to cerebrovascular damage and post-stroke progression [1,2]. Inflammatory mediators and immune-related pathways are increasingly recognized as potential mechanistic and therapeutic targets in ischemic stroke [3,4]. Identifying exposure-related inflammatory biomarkers may therefore help clarify disease susceptibility and provide new insight into stroke prevention and risk stratification.

Artificial sweeteners—including erythritol, aspartame, and sucralose—have been widely adopted as low- or non-caloric sugar substitutes [5,6]. Although these compounds are often promoted for weight management and glycemic control, their long-term cerebrovascular and inflammatory effects remain insufficiently defined [7]. These concerns are particularly consequential: the populations with the highest consumption of artificial sweeteners are disproportionately those already burdened by Cardiovascular-Kidney-Metabolic (CKM) syndrome—a state characterized by chronic low-grade inflammation and endothelial dysfunction that may render the cerebrovascular system especially vulnerable to additional inflammatory insults [8]. Although classical CKM risk factors (hypertension, dyslipidemia, and hyperglycemia) are well-established drivers of disease progression, they do not fully explain the observed heterogeneity in individual susceptibility [9]. This gap raises a clinically urgent question: Do ubiquitous artificial sweeteners constitute novel, independent exposures that accelerate CKM progression in this vulnerable population?

Despite accumulating interest, the safety profiles of individual sweeteners remain poorly defined. Several fundamental questions also persist. For instance, it is unclear whether distinct relationships exist between specific sweeteners and discrete CKM-related phenotypes. It is also unknown if adverse effects are broadly shared across erythritol, aspartame, and sucralose. Furthermore, conventional observational evidence has inherent limitations, including residual confounding and reverse causation [10,11]. These limitations make it difficult to establish whether any observed sweetener–outcome association is independent of established metabolic risk factors. Beyond simple association, the molecular mechanisms, immune microenvironmental alterations, and protein-level interactions remain uncharacterized.

To systematically address these gaps, we designed an integrated genetic and multi-omics framework. First, using two-sample Mendelian randomization (TSMR), we compared the associations of erythritol-, aspartame-, and sucralose-related genetic traits with a broad panel of CKM-related phenotypes and identified the strongest evidence of association between genetically predicted erythritol levels and stroke. Second, multivariable Mendelian randomization (MVMR) was used to evaluate whether this association persisted after adjustment for selected cardiometabolic traits. Third, downstream network, transcriptomic, machine-learning, immune-related, and docking analyses were used to prioritize stroke-related biological pathways and candidate genes within the predicted erythritol–stroke framework. These downstream analyses were exploratory and hypothesis-generating and were not designed to establish direct erythritol-induced molecular regulation or mediation. By integrating genetic association with downstream hypothesis-generating bioinformatic prioritization, this study provides a framework for investigating inflammatory and immune processes potentially relevant to erythritol-associated cerebrovascular risk. These findings raise concern regarding sustained elevations in circulating erythritol and support further evaluation of dietary exposures that increase circulating erythritol, although a direct causal effect of erythritol consumption was not established. Notably, genetically predicted circulating erythritol should not be considered equivalent to dietary erythritol intake.

## 2. Results

### 2.1. Two-Sample Mendelian Randomization Identifies an Association Between Genetically Predicted Circulating Erythritol and Stroke

To evaluate the potential impact of artificial sweeteners on CKM syndrome, TSMR analysis was performed. Three common sweeteners were investigated as exposures. These included erythritol, aspartame, and sucralose. They were systematically tested against 10 CKM-related phenotypes. All MR results were further validated by sensitivity analyses, and the detailed results are presented in Supplementary Tables S2 and S3 and Supplementary Figure S1.

**Figure 1:**
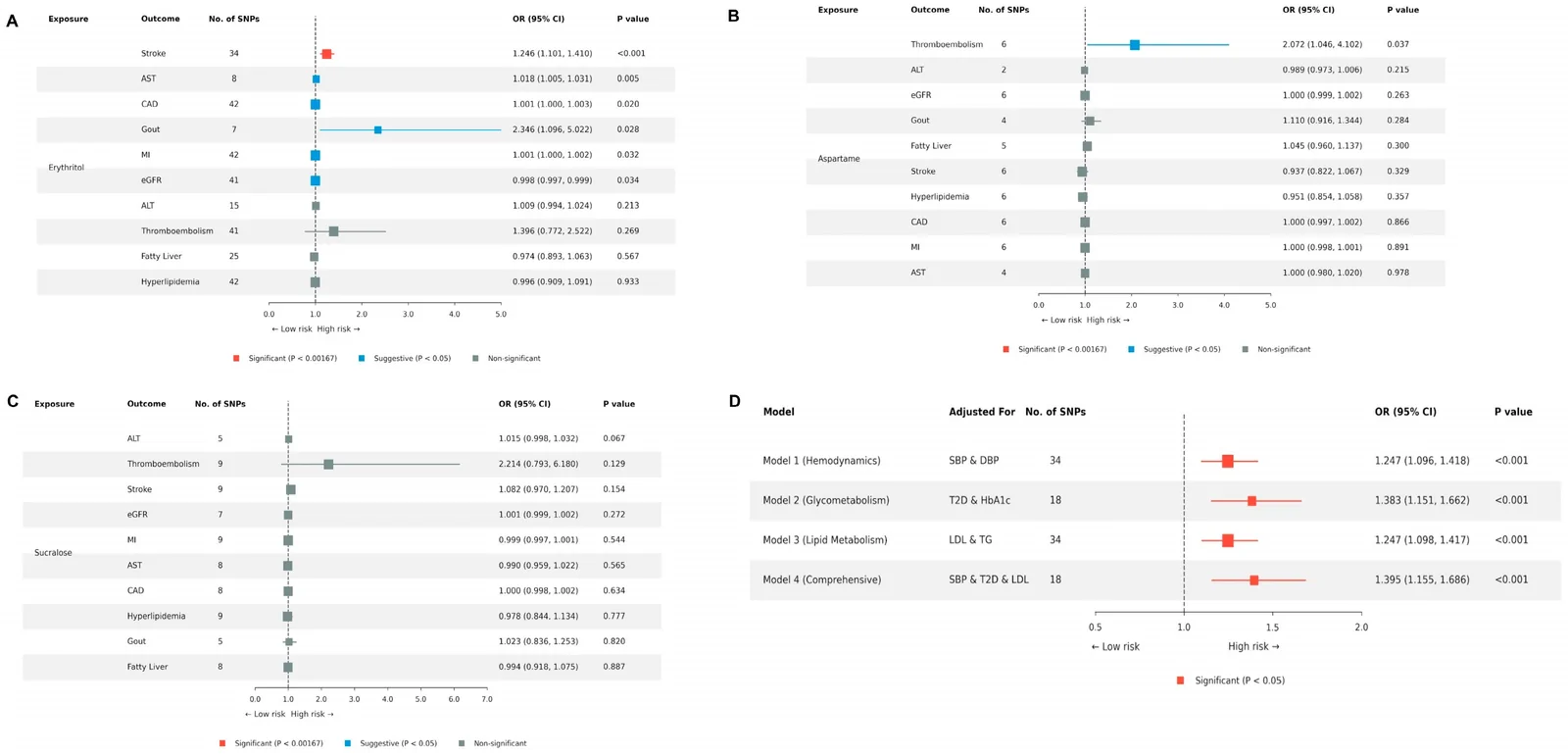
Mendelian randomization (MR) analyses of artificial sweeteners on CKM phenotypes. (**A**–**C**) Two-sample MR estimates for genetically predicted erythritol, aspartame, and sucralose across 10 cardiovascular–kidney–metabolic (CKM) outcomes. (**D**) Multivariable MR analysis of circulating erythritol and stroke after adjustment for blood pressure-, glycemic-, and lipid-related traits. Odds ratios (ORs) and 95% confidence intervals (CIs) are shown. Red, blue, and grey squares indicate Bonferroni-significant (*p* < 0.00167), nominally significant (*p* < 0.05), and non-significant associations, respectively. Among the tested sweetener-related traits, genetically predicted circulating erythritol showed the strongest association with stroke, which remained significant after adjustment for selected cardiometabolic traits.

The TSMR results for erythritol are illustrated in Figure 1A. Genetically predicted circulating erythritol levels showed a strong positive association with stroke risk (OR = 1.246, 95% CI: 1.101–1.410, *p* < 0.001). Crucially, this core finding successfully crossed the strict Bonferroni-corrected significance threshold (*p* < 0.00167). Several other traits, including AST, CAD, gout, MI, and eGFR, demonstrated only suggestive associations (*p* < 0.05). Consequently, stroke emerged as the most prominent and reliable CKM target of erythritol.

Parallel analyses were conducted for the other two sweeteners. The forest plot for aspartame (Figure 1B) showed a suggestive association with thromboembolism, but no significance for stroke. Similarly, univariable MR for sucralose (Figure 1C) revealed no statistically significant associations with any CKM outcomes. These comparative results highlight a comparatively stronger association. Among the tested sweetener-related circulating traits, genetically predicted erythritol showed the strongest evidence of association with stroke.

### 2.2. Multivariable MR Shows That the Association Between Genetically Predicted Circulating Erythritol and Stroke Persists After Adjustment for Selected Cardiometabolic Traits

To assess whether the association between genetically predicted erythritol levels and stroke persisted after adjustment for selected CKM-related risk factors, multivariable Mendelian randomization was performed with adjustment for hemodynamic, glycometabolic, and lipid-related traits (Supplementary Table S4). As shown in Figure 1D, the association between genetically predicted erythritol levels and stroke remained robust across all multivariable models. Harmonization and sensitivity analyses evaluating heterogeneity and pleiotropy confirmed model robustness (Supplementary Table S5).

After adjustment for systolic and diastolic blood pressure, erythritol remained significantly associated with increased stroke risk (OR = 1.247, 95% CI: 1.096–1.418, *p* < 0.001). This association was also preserved after adjustment for type 2 diabetes and HbA1c (OR = 1.383, 95% CI: 1.151–1.662, *p* < 0.001), as well as after adjustment for LDL cholesterol and triglycerides (OR = 1.247, 95% CI: 1.098–1.417, *p* < 0.001). In the comprehensive model incorporating systolic blood pressure, type 2 diabetes, and LDL cholesterol, the association remained significant and directionally consistent (OR = 1.395, 95% CI: 1.155–1.686, *p* < 0.001).

Together, these findings indicate that the observed association between genetically predicted erythritol levels and stroke was not fully explained by the selected blood pressure-, glycemic-, and lipid-related traits included in the MVMR models.

### 2.3. Identification of Common Targets Between Erythritol and Stroke

**Figure 2:**
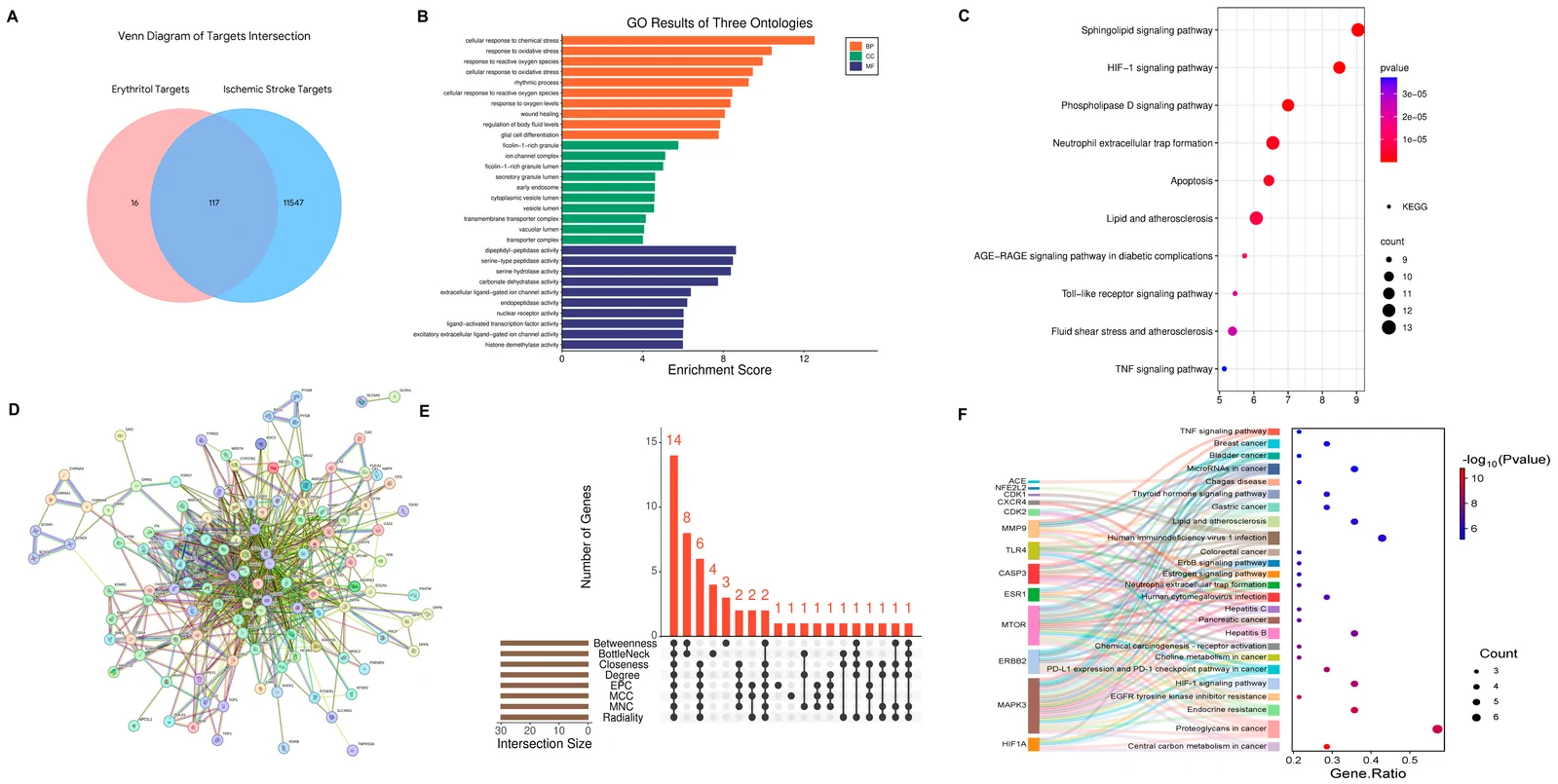
Identification and functional prioritization of candidate genes within the predicted erythritol–stroke overlap. Enrichment and network analyses were used to characterize the biological processes represented among the overlapping candidate genes and to prioritize genes for subsequent transcriptomic and machine-learning analyses. (**A**) Overlap between 133 predicted erythritol targets and stroke-associated genes, identifying 117 shared candidates. (**B**,**C**) GO and KEGG enrichment analyses of the overlapping genes. (**D**) Protein–protein interaction network of the 117 candidates. (**E**) Integration of multiple network-topology algorithms to prioritize hub genes. (**F**) Pathway distribution of the prioritized hub genes. Overall, the overlapping candidates converged on stroke-related inflammatory, oxidative-stress, hypoxic, and vascular-injury pathways, providing hypothesis-generating targets for subsequent analyses.

To identify potential molecular targets linking erythritol exposure to stroke, 133 erythritol-related targets predicted by SwissTargetPrediction and SuperPred were intersected with 11,664 stroke-associated genes from GeneCards. A total of 117 overlapping erythritol–stroke cross-targets were identified and used for subsequent enrichment and network analyses (Figure 2A; Supplementary Tables S6 and S7). Using 19,273 HGNC-approved human protein-coding genes as the background universe, the 117 observed overlapping genes exceeded the 59.63 expected by chance, corresponding to a 1.96-fold enrichment (one-sided hypergeometric *p* = 1.82 × 10^−25^). In a post hoc sensitivity analysis using the median GeneCards Relevance Score of 0.7874, 4876 protein-coding stroke-associated genes were retained, of which 96 overlapped with the 133 predicted erythritol targets. This overlap also exceeded chance expectations (33.65 genes; 2.85-fold enrichment; *p* = 7.54 × 10^−30^), and all 14 PPI-prioritized genes were retained. These results indicate that the observed overlap exceeded chance expectations and was not driven solely by low-relevance GeneCards entries. However, the overlap and enrichment analyses were used only for candidate prioritization and hypothesis generation and should not be interpreted as independent evidence of an erythritol-specific molecular mechanism. Accordingly, the subsequent enrichment, network, transcriptomic, immune-related, and docking analyses were interpreted as exploratory and hypothesis-generating and were not considered evidence of direct erythritol-induced molecular regulation or mediation.

### 2.4. Functional Enrichment Analysis of the Intersecting Targets

Functional enrichment of the 117 intersecting genes highlighted key mechanisms in stroke pathogenesis. GO analysis (Figure 2B) revealed a strong association with oxidative stress and tissue injury, particularly “response to reactive oxygen species” and “neuron death.” After filtering out generic pan-cancer pathways, KEGG analysis (Figure 2C) demonstrated significant enrichment in classical cerebrovascular cascades, including “Lipid and atherosclerosis,” “HIF-1 signaling,” and “Apoptosis.” Collectively, these enrichment results highlight oxidative stress, endothelial dysfunction, and vascular injury as stroke-related biological processes represented among the overlapping candidate genes. These findings are exploratory and do not establish that erythritol directly induces these molecular changes.

### 2.5. Construction of the PPI Network and Hub Genes Analysis

A PPI network was constructed using the 117 erythritol–stroke cross-targets, revealing extensive interactions among these genes (Figure 2D). Hub genes were then screened using multiple topological algorithms and integrated by UpSet analysis (Figure 2E). Pathway analysis of these hub genes highlighted several stroke-related pathways, including HIF-1 signaling, Toll-like receptor signaling, apoptosis, lipid and atherosclerosis, fluid shear stress and atherosclerosis, TNF signaling, and neutrophil extracellular trap formation (Figure 2F). These results indicate that the prioritized overlapping candidate genes are enriched in biological processes related to inflammation, oxidative stress, endothelial dysfunction, and vascular injury in stroke.

### 2.6. Machine-Learning Prioritization of Stroke-Related Candidate Genes

**Figure 3:**
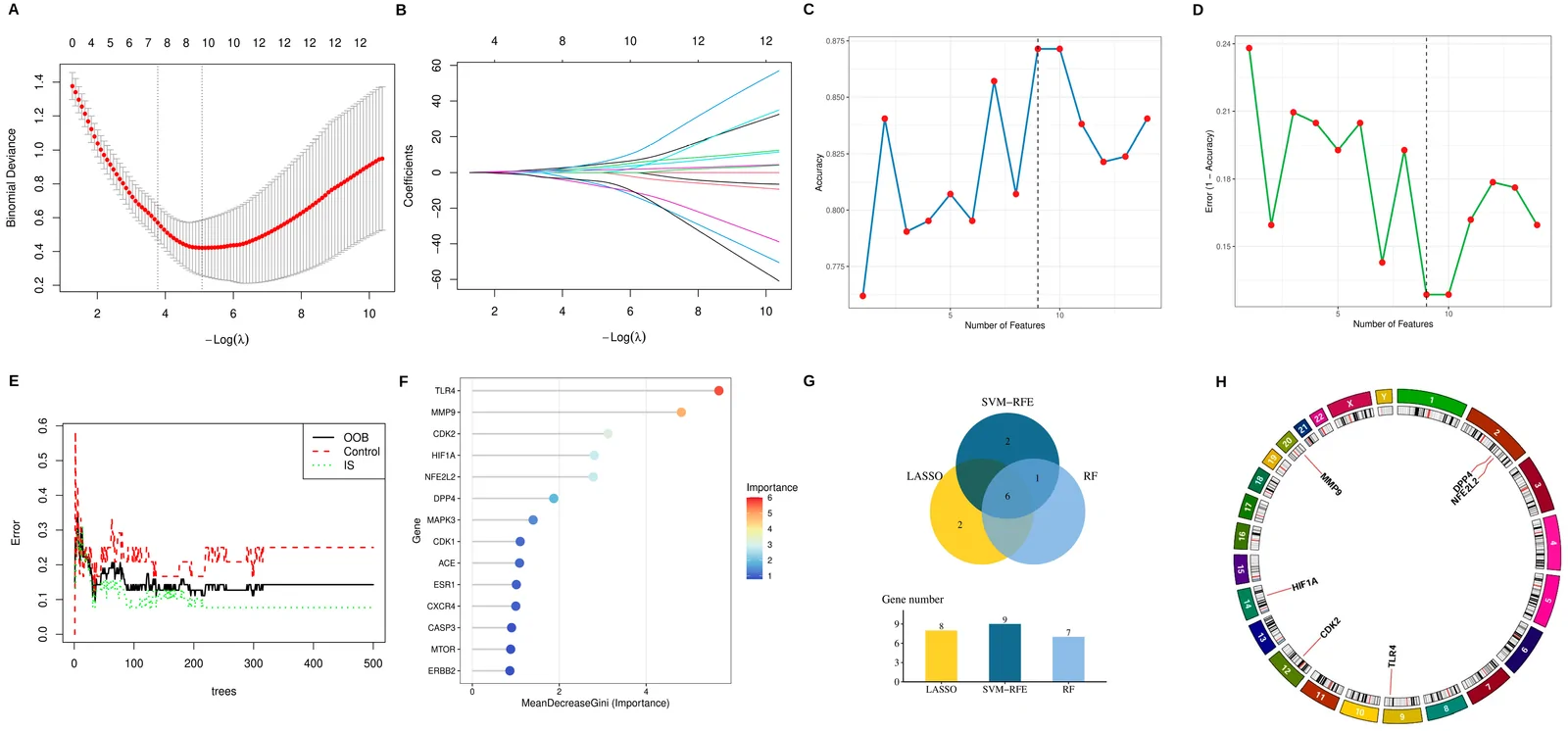
Multi-algorithm prioritization of stroke-related candidate genes. Three complementary feature-selection approaches were used to identify candidate genes that consistently contributed to the discrimination of ischemic stroke and control samples. (**A**,**B**) LASSO regression selected eight candidate genes based on cross-validation and coefficient shrinkage. (**C**,**D**) SVM-RFE identified nine candidate genes according to cross-validation accuracy and error. (**E**,**F**) Random Forest analysis prioritized seven genes based on model error and feature importance. (**G**) Integration of the three approaches identified six consensus genes: HIF1A, TLR4, MMP9, NFE2L2, DPP4, and CDK2. (**H**) Chromosomal locations of the six consensus genes. The convergence of three complementary feature-selection approaches prioritized six genes for subsequent classification and interpretation analyses.

Advanced analysis of the 14 hub genes was performed using three machine learning approaches: LASSO regression (Figure 3A,B), SVM-RFE (Figure 3C,D), and Random Forest (Figure 3E,F). Integration of results from these algorithms (Figure 3G) identified six consensus genes—HIF1A, TLR4, MMP9, NFE2L2, DPP4, and CDK2—as candidate genes associated with stroke classification within the predicted erythritol—stroke overlap. Additionally, circular graphs were generated to visualize the specific chromosomal locations of these six core genes (Figure 3H).

### 2.7. Leakage-Resistant Nested Cross-Validation Supports Model Robustness

To further evaluate the robustness of the machine-learning findings while minimizing potential information leakage, an additional repeated nested cross-validation analysis was performed using the 14 prespecified candidate genes. All preprocessing, feature selection, hyperparameter tuning, and model fitting were conducted exclusively within each outer training fold, while the corresponding held-out samples remained unseen until model evaluation. Thus, information from the validation folds did not contribute to gene selection or model optimization.

Across 10 repeats of 5-fold outer cross-validation, the mean outer AUC was 0.935 ± 0.032, and the pooled out-of-fold AUC was 0.960 (95% CI, 0.912–1.000; Supplementary Figure S2A). Feature-selection stability analysis showed that MMP9 was selected in 100% of outer training folds, whereas HIF1A and TLR4 were each selected in 92% (Supplementary Figure S2B), indicating stable prioritization across repeated data partitions. Notably, these three genes were also prioritized in the original multi-algorithm workflow and incorporated into the subsequent three-gene logistic model, providing an additional robustness check for their prioritization.

The final LASSO model was subsequently locked and applied unchanged to the independent GSE58294 cohort, yielding an AUC of 0.802 (95% CI, 0.711–0.892; Supplementary Figure S2C). No external samples were used for preprocessing estimation, feature selection, hyperparameter tuning, or model refitting. These findings support the reproducibility of the stroke-related expression signal while reducing the likelihood that the observed performance was driven by information leakage or a single favorable partition of the training cohort.

### 2.8. Predictive Model Construction and SHAP Value Interpretation

**Figure 4:**
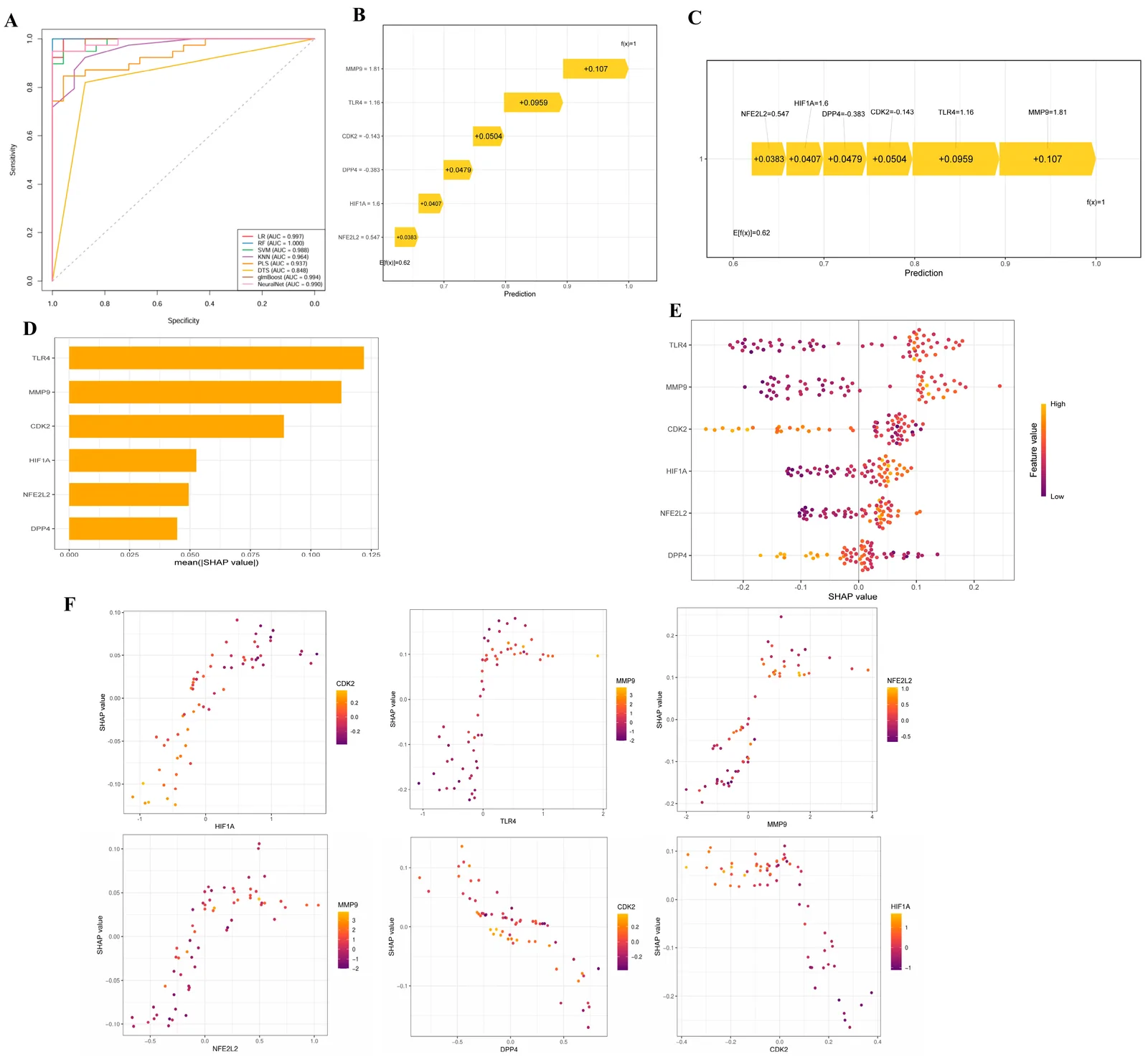
Comparison and interpretation of machine-learning models for ischemic stroke classification. Eight supervised classifiers were compared to evaluate the ability of the prioritized candidate genes to distinguish ischemic stroke from control samples, while SHAP analysis was used to interpret the contribution of individual genes to model predictions. (**A**) ROC curves comparing eight supervised classifiers based on the prioritized candidate genes. (**B**,**C**) Representative SHAP waterfall and force plots illustrating gene contributions to individual predictions. (**D**,**E**) Global SHAP feature importance and summary plots showing the relative contribution and direction of gene effects. (**F**) Gene-specific SHAP dependence plots showing relationships between gene expression and model contribution. The random forest model showed the highest discriminatory performance, while SHAP analysis identified heterogeneous contributions of the six candidate genes, with MMP9 and TLR4 among the strongest contributors.

Eight supervised machine learning models, including PLS, LR, glmBoost, RF, DTS, SVM, neural network, and KNN, were constructed to evaluate the predictive performance for ischemic stroke classification. Among these models, the RF model showed the highest AUC and was therefore selected for further interpretation (Figure 4A). SHAP analysis was subsequently performed to quantify the contribution of the six core genes to the RF model. Waterfall and force plots illustrated the cumulative effects of individual genes on the prediction score in representative samples (Figure 4B,C). The SHAP bar plot showed that MMP9, TLR4, HIF1A, CDK2, NFE2L2, and DPP4 contributed to the RF model to varying degrees, with MMP9 and TLR4 showing the strongest effects and DPP4 showing the weakest influence (Figure 4D). The SHAP summary plot further demonstrated heterogeneous gene-specific effects on sample classification, suggesting that these core genes collectively contributed to the predictive performance of the RF model (Figure 4E). Gene-specific SHAP scatter plots revealed nonlinear relationships between gene expression levels and prediction contribution (Figure 4F). These findings provided an interpretable basis for subsequent refinement of candidate predictors using multivariable logistic regression.

After machine learning-based feature screening, multivariable logistic regression was further applied to refine the candidate stroke-related molecular signature. Although SHAP analysis identified several important contributors to the RF model, machine learning algorithms mainly capture complex nonlinear patterns that may not be directly transferable to a clinically interpretable linear prediction model. Therefore, to improve model stability and clinical applicability, candidate predictors were further evaluated in a multivariable LR framework. Inclusion of all top-ranked SHAP features resulted in potential multicollinearity and overfitting, particularly reflected by unstable estimates for CDK2. After feature refinement, a simplified three-gene signature consisting of MMP9, TLR4, and HIF1A was established.

### 2.9. Construction and Validation of a Three-Gene Stroke Classification Model

**Figure 5:**
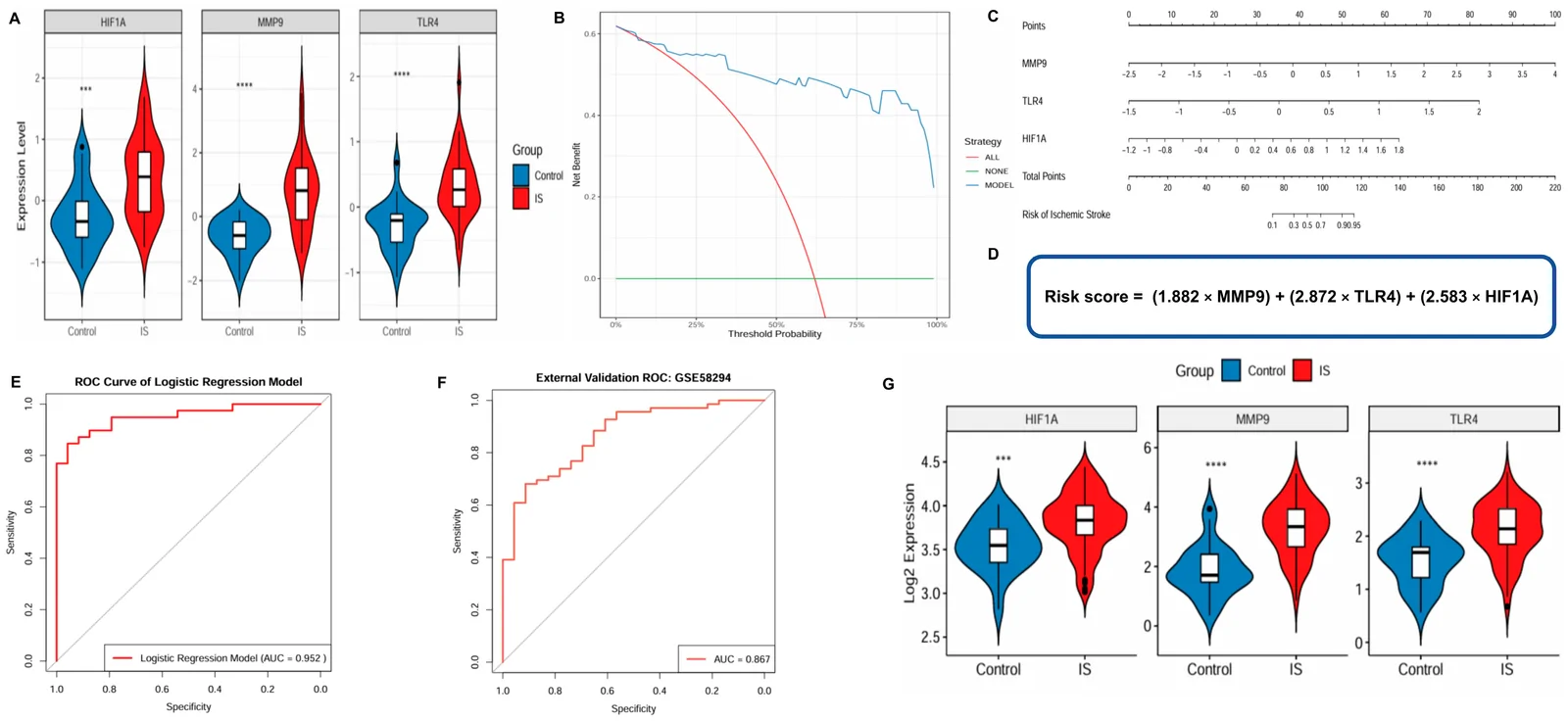
Construction and classification validation of a three-gene stroke-related classification model. This analysis evaluated whether the three prioritized genes could jointly discriminate stroke from control samples and whether their discriminatory performance was reproducible in an independent transcriptomic cohort. (**A**) Expression of HIF1A, MMP9, and TLR4 in ischemic stroke and control samples in GSE16561. (**B**,**C**) Decision curve analysis and nomogram of the three-gene model. (**D**) Logistic regression classification score formula based on MMP9, TLR4, and HIF1A. (**E**) ROC curve in the training cohort. (**F**) External ROC analysis in GSE58294. (**G**) Expression of the three genes in the external validation cohort. The three-gene model showed reproducible discrimination between ischemic stroke and control samples across the training and external cohorts. *** *p* < 0.001; **** *p* < 0.0001.

The three selected genes were significantly upregulated in ischemic stroke samples compared with controls (Figure 5A). Based on the multivariable LR coefficients, a classification score was calculated as follows: classification score = (1.882 × MMP9) + (2.872 × TLR4) + (2.583 × HIF1A) (Figure 5D). A nomogram was constructed to facilitate visualization of the three-gene classification model (Figure 5C), and decision curve analysis indicated favorable clinical net benefit across a broad range of threshold probabilities (Figure 5B). The classification model showed strong discriminatory performance in the training dataset, with an AUC of 0.952 (Figure 5E). External validation in GSE58294 further confirmed its classification performance, yielding an AUC of 0.867 (Figure 5F). Consistently, MMP9, TLR4, and HIF1A were also significantly elevated in the validation dataset (Figure 5G), supporting the reliability and generalizability of the three-gene candidate molecular signature.

### 2.10. GSVA Enrichment, Immune Infiltration, and Immunocyte Correlation Analysis

**Figure 6:**
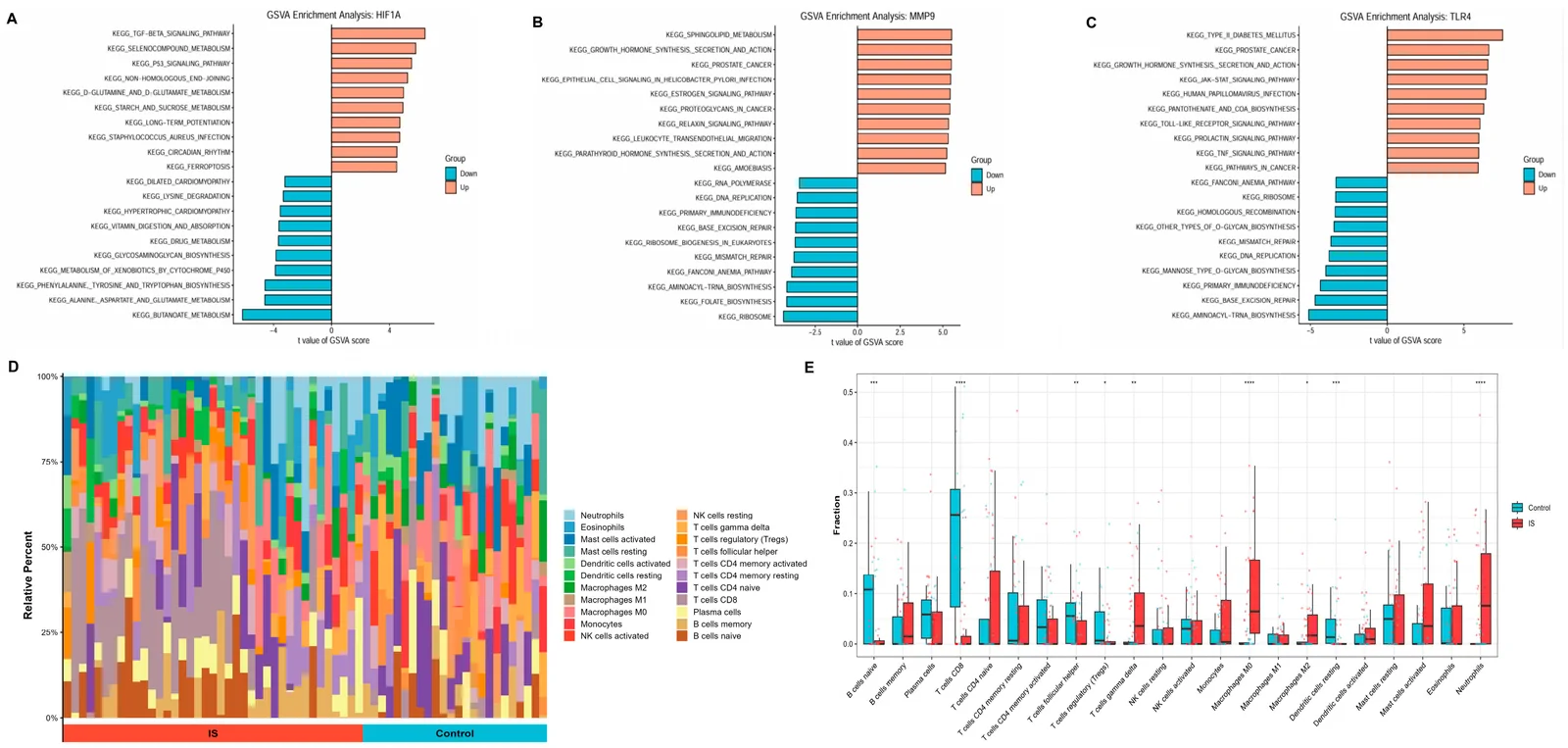
Pathway and immune-cell characteristics associated with the three prioritized stroke-related candidate genes. GSVA and CIBERSORT analyses were used to characterize biological pathways associated with HIF1A, MMP9, and TLR4 and to assess differences in immune-cell composition between ischemic stroke and control samples. (**A**–**C**) GSVA showing KEGG pathways associated with HIF1A, MMP9, and TLR4, respectively. (**D**) CIBERSORT-estimated proportions of 22 immune-cell subsets across ischemic stroke (IS) and control samples. (**E**) Comparison of immune-cell fractions between the two groups. These analyses indicate that the three candidate genes are associated with inflammatory, stress-related, and metabolic pathways, accompanied by alterations in the immune-cell composition of ischemic stroke samples. Blue indicates controls and red indicates IS samples. Asterisks indicate statistically significant differences between groups: * *p* < 0.05, ** *p* < 0.01, *** *p* < 0.001, **** *p* < 0.0001.

To investigate the biological relevance of the three prioritized stroke-related candidate genes, GSVA was performed for HIF1A, MMP9, and TLR4. These genes were associated with multiple KEGG pathways related to immune regulation, inflammatory signaling, cellular stress, and metabolic processes. Representative pathways included TGF-β and p53 signaling for HIF1A, sphingolipid metabolism and leukocyte transendothelial migration for MMP9, and JAK–STAT, Toll-like receptor, TNF signaling, and apoptosis-related pathways for TLR4 (Figure 6A–C).

**Figure 7:**
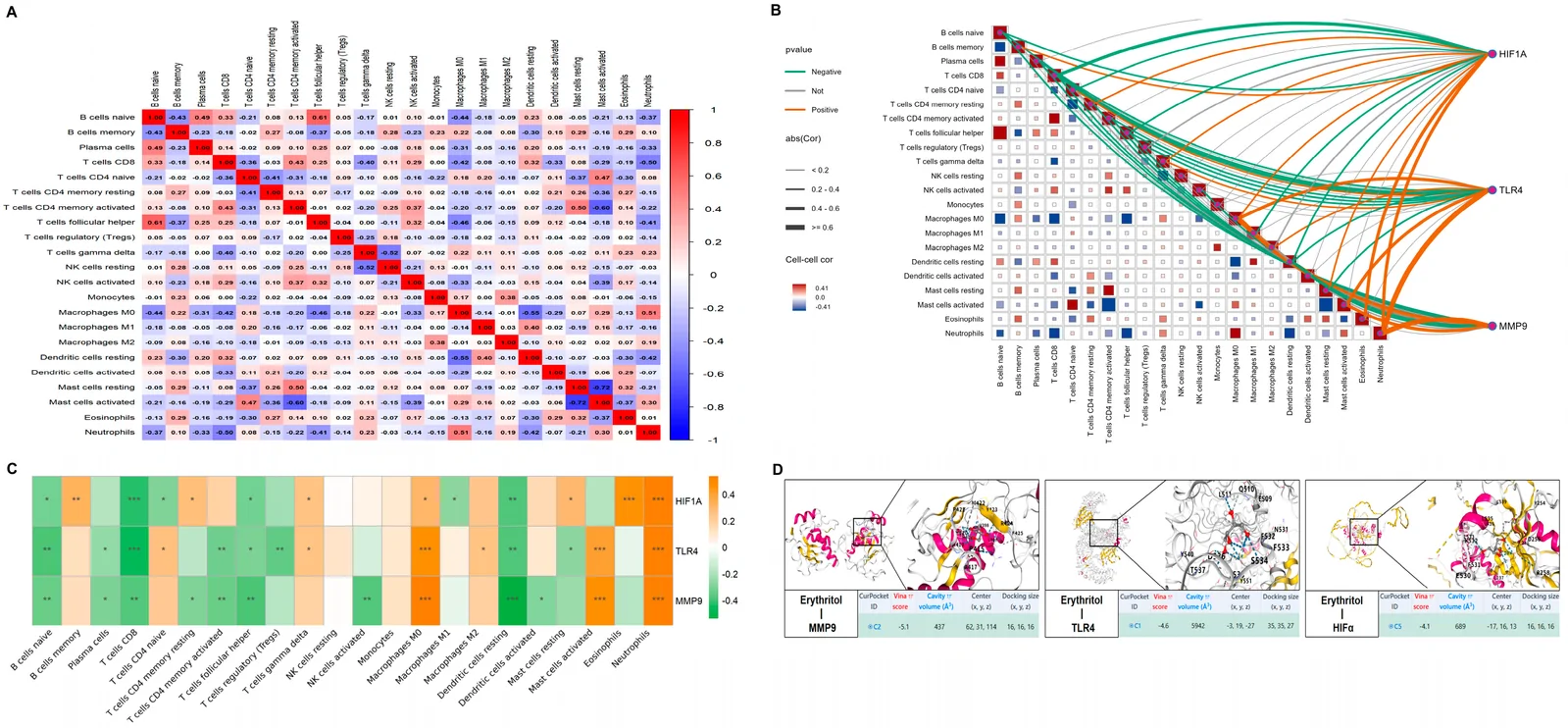
Immune-cell associations and exploratory molecular docking of the three prioritized stroke-related candidate genes. Correlation analysis was used to examine the relationships between the three candidate genes and immune-cell infiltration, whereas molecular docking was performed as an exploratory structural analysis of computationally predicted erythritol–protein poses. (**A**–**C**) Correlations of MMP9, TLR4, and HIF1A with infiltrating immune-cell subsets; symbol color and size represent the direction and strength of the correlations. (**D**) Computationally predicted docking poses of erythritol with MMP9, TLR4, and HIF1A. The correlation analyses highlight associations between the three genes and the immune microenvironment of ischemic stroke, whereas the docking results represent exploratory structural predictions and do not establish direct or high-affinity erythritol–protein binding. * *p* < 0.05; ** *p* < 0.01; *** *p* < 0.001.

CIBERSORT was then used to estimate the proportions of 22 immune cell subsets in ischemic stroke and control samples. The immune infiltration landscape showed marked inter-sample heterogeneity, and several immune cell subsets differed significantly between groups (Figure 6D,E). Further correlation analysis revealed complex relationships among immune cell populations and showed that HIF1A, MMP9, and TLR4 were associated with multiple immune cell subsets, including T cells, macrophages, dendritic cells, eosinophils, and neutrophils (Figure 7A–C). These findings indicate that the three prioritized candidate genes are associated with pathway alterations and immune-cell patterns in stroke; however, these transcriptomic analyses do not establish erythritol-specific regulation.

### 2.11. Molecular Docking

Molecular docking analysis showed that erythritol could be docked into the predicted binding pockets of MMP9, TLR4, and HIF1A, with binding energies below −4 kcal/mol, suggesting thermodynamically favorable but relatively weak interactions (Figure 7D). Considering the small, highly hydrophilic, and polyhydroxylated structure of erythritol, it is unlikely to behave as a typical high-affinity drug-like ligand. Thus, the docking results should be interpreted as exploratory structural evidence. These findings suggest possible molecular contacts between erythritol and the three proteins.

## 3. Discussion

In the present study, we integrated genetic epidemiology, multivariable Mendelian randomization, network-based target prediction, transcriptomic analysis, machine learning, immune infiltration analysis, and molecular docking to systematically investigate the relationship between multiple artificial sweeteners and CKM-related phenotypes. Among the three artificial sweeteners evaluated, genetically predicted circulating erythritol showed the strongest association with stroke. This association remained significant after adjustment for representative hemodynamic, glycometabolic, and lipid-related traits in multivariable MR models, suggesting that the erythritol–stroke relationship may not be fully explained by traditional CKM risk factors. Further multi-omics analyses identified a simplified three-gene signature consisting of MMP9, TLR4, and HIF1A, which showed discriminatory value for ischemic stroke and was closely related to inflammatory signaling, oxidative stress, immune remodeling, and vascular injury. Together, these findings provide a genetically informed framework linking elevated circulating erythritol with stroke susceptibility, while not directly evaluating dietary erythritol intake.

Erythritol is widely used as a low-calorie sugar substitute and has long been considered metabolically inert because of its minimal short-term effects on blood glucose and insulin levels [12]. However, its increasing consumption in sugar-free and low-calorie products has raised concerns regarding its long-term cardiometabolic safety, particularly among individuals with obesity, diabetes, and high cardiovascular risk [13,14]. Conventional observational studies in this field are prone to residual confounding and reverse causality, as individuals with poor metabolic status may be more likely to consume artificial sweeteners before the development or diagnosis of cardiovascular disease [15]. Therefore, MR provides a valuable complementary approach by using genetic variants as instrumental variables to reduce confounding and reverse causation [11,16]. In our TSMR analysis, erythritol displayed a positive association with stroke, whereas the evidence for other sweeteners and CKM-related outcomes was comparatively weaker or nonsignificant. Importantly, the association between genetically predicted erythritol levels and stroke persisted in MVMR models adjusting for blood pressure, glycometabolic traits, and lipid-related traits, indicating that the association was not fully explained by the specific cardiometabolic traits included in these models [17,18]. An important interpretive distinction is that the exposure evaluated in our MR analysis represents genetically predicted circulating erythritol rather than dietary erythritol intake. Circulating erythritol concentrations may reflect exogenous ingestion as well as endogenous production, metabolic processing, and renal handling [19,20]. Therefore, the present findings should not be interpreted as direct evidence that consuming erythritol-containing foods increases stroke risk. Nevertheless, because dietary erythritol can markedly increase circulating erythritol concentrations, the observed association raises a potential safety signal regarding high or sustained erythritol consumption, particularly in individuals at elevated cardiovascular risk. Dedicated prospective and interventional studies are required to determine the specific effects of dietary erythritol exposure.

Experimental and human ingestion studies have also raised the possibility that dietary erythritol, through acute increases in circulating erythritol, may influence vascular or thrombotic processes [14]. Erythritol has been reported to enhance platelet reactivity and thrombus formation, which may increase thrombotic susceptibility [14]. Other evidence suggests that erythritol may influence macrophage function and inflammatory responses, while long-term exposure to artificial sweeteners may impair endothelial progenitor cell function and angiogenesis in ischemic brain regions [21,22]. These mechanisms are broadly consistent with our enrichment analyses, which highlighted pathways related to HIF-1 signaling, Toll-like receptor signaling, TNF signaling, neutrophil extracellular trap formation, apoptosis, lipid and atherosclerosis, and fluid shear stress and atherosclerosis [23,24]. These pathway-level findings are consistent with biological processes previously implicated in stroke, including inflammation, endothelial dysfunction, oxidative stress, and vascular injury. However, the present bioinformatic analyses do not demonstrate that erythritol directly induces these processes.

Through PPI network analysis, machine learning, SHAP interpretation, and multivariable LR refinement, MMP9, TLR4, and HIF1A were prioritized as a compact stroke-related gene signature within the predicted erythritol–stroke candidate framework. TLR4 is a key mediator of innate immune activation and inflammatory signaling, and its activation can amplify downstream NF-κB- and cytokine-related responses [25,26,27]. MMP9 is closely involved in extracellular matrix remodeling, blood–brain barrier disruption, leukocyte migration, and post-ischemic vascular injury [28,29]. HIF1A is a central hypoxia-responsive regulator that participates in ischemic adaptation, angiogenesis, inflammatory metabolism, and cellular stress responses [30,31]. Although TLR4, MMP9, and HIF1A are involved in distinct biological processes, their dysregulation may converge on interconnected pathways in ischemic stroke. TLR4-mediated innate immune activation may promote inflammation and endothelial dysfunction, while MMP9-mediated extracellular matrix degradation can impair blood–brain barrier integrity and facilitate edema and secondary tissue injury [25,32]. Meanwhile, HIF1A-dependent hypoxic responses interact with oxidative stress and inflammatory signaling [33]. These processes are unlikely to act independently. Innate immune activation and oxidative stress may aggravate endothelial dysfunction, whereas disruption of the extracellular matrix and blood–brain barrier can facilitate leukocyte infiltration and further amplify local inflammation [34]. Together with hypoxia-driven cellular stress, these mutually reinforcing processes may promote microvascular dysfunction and a prothrombotic vascular environment during the acute phase of ischemic stroke, thereby contributing to lesion expansion and secondary neuronal damage. Their cumulative effects may ultimately influence infarct evolution and neurological outcome. The convergence of TLR4-, MMP9-, and HIF1A-related pathways provides a biologically plausible framework linking immune activation, neurovascular injury, and hypoxic stress across stroke development and progression. Thus, the three-gene signature may reflect an integrated immune–vascular–hypoxic injury axis relevant to ischemic stroke. However, all three genes are established components of general stroke pathophysiology independent of erythritol exposure; their prioritization should therefore be regarded as hypothesis-generating rather than evidence of an erythritol-specific molecular mechanism. Moreover, because the initial erythritol targets were computationally predicted and the GeneCards stroke-gene set was intentionally broad, the observed overlap and its statistical enrichment should be regarded as a candidate-prioritization step rather than independent mechanistic evidence. The downstream analyses therefore remain exploratory and hypothesis-generating and do not establish direct erythritol–protein interactions or molecular mediation.

Although SHAP analysis highlighted several contributors to the RF model, machine learning algorithms mainly capture nonlinear and interaction-based patterns that may not be directly transferable to clinically interpretable linear models [35]. During multivariable LR refinement, inclusion of all top-ranked SHAP features led to potential model instability and overfitting, particularly reflected by unstable estimates for CDK2. Therefore, the final model retained MMP9, TLR4, and HIF1A, balancing predictive performance, biological interpretability, and clinical simplicity. The resulting three-gene model retained good discriminatory ability in the external validation dataset, supporting the reproducibility of the stroke-related expression signature. From a clinical perspective, the MR and transcriptomic findings should be interpreted at different levels: the MR analysis identifies genetically predicted circulating erythritol as a potential cerebrovascular risk signal warranting prospective evaluation, whereas the three-gene signature primarily reflects stroke-related molecular alterations and may have potential value for stroke classification.

GSVA and immune infiltration analyses further supported the relevance of the three-gene signature to inflammatory, immune, stress-related, and metabolic processes in ischemic stroke [36,37]. CIBERSORT and correlation analyses also identified associations between the three genes and multiple immune-cell subsets. Because the transcriptomic datasets contained no erythritol measurements, these findings reflect general stroke-related biology rather than erythritol-specific pathway or immune regulation. Molecular docking generated predicted erythritol–protein poses for MMP9, TLR4, and HIF1A [38,39,40]. However, given the small, highly hydrophilic, and polyhydroxylated structure of erythritol, these docking results should be interpreted only as exploratory structural observations and cannot establish specific binding, target engagement, or functional regulation. Biochemical and functional validation is therefore required.

This study has several strengths. First, the integration of TSMR and MVMR provided a genetically informed framework to evaluate the relationship between artificial sweeteners and CKM-related phenotypes while reducing confounding and reverse causality. Second, the analysis covered multiple artificial sweeteners and multiple clinically relevant outcomes, allowing comparison across a broader CKM spectrum. Third, the combination of target prediction, transcriptomic analysis, machine learning, SHAP interpretation, immune infiltration analysis, and molecular docking provided a multidimensional, hypothesis-generating framework for prioritizing stroke-related pathways and candidate genes potentially relevant to the observed erythritol–stroke association. Finally, external validation of the simplified three-gene model provided additional evidence for reproducible stroke classification across independent transcriptomic cohorts.

Several limitations should be noted. First, most GWAS datasets included in the MR analyses were derived from individuals of European ancestry; therefore, the generalizability of our findings to other populations requires further validation. Additionally, the genetic instruments used in this study represent circulating erythritol concentrations and cannot distinguish whether these concentrations arise from dietary intake, endogenous production, metabolic variation, or renal handling. Second, the multi-omics and immune infiltration analyses were mainly based on transcriptomic datasets that contained no measurements of erythritol exposure or circulating erythritol levels. Therefore, these analyses should be considered exploratory and hypothesis-generating, and they cannot establish erythritol-specific gene regulation or molecular mediation. Experimental studies are required to determine whether erythritol directly affects MMP9, TLR4, HIF1A, oxidative stress, endothelial function, or related vascular pathways. Third, molecular docking only provides preliminary structural evidence, and future biochemical studies are warranted to verify the potential interactions between erythritol and the identified proteins.

## 4. Methods

### 4.1. Two-Sample Mendelian Randomization (TSMR)

This survey mainly utilized publicly available summary-level data from the European population, which originated from the OpenGWAS database project that integrates epidemiological units [16]. A study from the European Bioinformatics Institute (EBI) database has obtained the GWAS summary statistics for genetically predicted circulating erythritol levels [41,42]. TSMR was performed to evaluate the potential causal associations of three artificial sweetener-related traits, including erythritol, aspartame, and sucralose, with 10 CKM-related phenotypes. Genetic instruments were selected using exposure-specific significance criteria, with detailed selection thresholds provided in Supplementary Table S1. After linkage-disequilibrium clumping using a 10,000-kb window and an r^2^ threshold of 0.001, 50, 9, and 6 independent SNPs were initially retained for erythritol, sucralose, and aspartame, respectively. The F-statistics ranged from 29.88 to 71.62 for erythritol, 21.48 to 25.29 for sucralose, and 16.00 to 25.00 for aspartame, with all values exceeding 10, indicating adequate instrument strength. After harmonization with the ischemic stroke outcome dataset, 34, 9, and 6 SNPs contributed to the IVW analyses for erythritol, sucralose, and aspartame, respectively. Heterogeneity and horizontal pleiotropy were assessed using Cochran’s Q statistic and the MR-Egger intercept test. Detailed instrument-selection criteria, SNP-level characteristics, and complete sensitivity-analysis results are provided in the Supplementary Tables. Specific details for each GWAS dataset, including sample sizes, the number of SNPs, unique GWAS identifiers, and data release years, are provided in Supplementary Table S1. Statistical significance was determined using a strict Bonferroni correction to account for multiple testing (*p* < 0.00167).

### 4.2. Multivariable Mendelian Randomization (MVMR)

Multivariable Mendelian randomization (MVMR) was conducted to evaluate whether the association between genetically predicted erythritol levels and ischemic stroke persisted after adjustment for selected established risk factors. Four sequential models were constructed, including three single-adjustment models (adjusting for blood pressure, glycemic traits, and lipid traits, respectively) and one combined model adjusting for all aforementioned risk factors. Genetic instruments were selected following the same criteria as those applied in the TSMR. Instrument harmonization and comprehensive sensitivity analyses—including evaluations of heterogeneity and pleiotropy—were performed to verify the robustness of the results.

### 4.3. Prediction of Erythritol Targets and Statistical Evaluation of Their Overlap with Stroke-Associated Genes

The canonical SMILES string of erythritol, C(C(C(CO)O)O)O, was obtained from PubChem and used to predict potential human protein targets using SwissTargetPrediction and SuperPred. The targets retrieved from the two databases were standardized to official gene symbols, combined, and deduplicated, yielding 133 unique predicted erythritol targets.

Stroke-associated genes were retrieved from GeneCards using the search term “ischemic stroke” on 28 March 2026. No Relevance Score cutoff was applied at the initial candidate-screening stage because this step was designed as a broad, sensitivity-oriented screen to minimize the premature exclusion of potentially relevant stroke-associated genes. All 11,664 unique entries returned in the GeneCards export were therefore retained as an initial candidate pool rather than being considered a high-confidence or experimentally validated stroke-gene set.

Because SwissTargetPrediction and SuperPred primarily return protein targets, the formal chance-overlap analysis was conducted within a protein-coding gene universe. All 117 overlapping genes remained within this protein-coding subset. To determine whether the observed overlap exceeded chance expectations, a one-sided hypergeometric test was performed using 19,273 HGNC-approved human protein-coding genes as the fixed background universe. The expected overlap was calculated as the product of the two gene-set sizes divided by the background-universe size, and fold enrichment was calculated as the observed overlap divided by the expected overlap.

To evaluate whether the observed enrichment was primarily driven by GeneCards entries with low Relevance Scores, an additional post hoc threshold-sensitivity analysis was conducted using the median Relevance Score of the complete exported GeneCards dataset as an objective distribution-based cutoff. The median Relevance Score was 0.7874. Protein-coding genes with Relevance Scores ≥ 0.7874 were retained, and the overlap, expected overlap, fold enrichment, hypergeometric *p* value, and odds ratio were recalculated using the same background universe. The overlapping genes were subsequently subjected to sequential candidate-prioritization procedures, including STRING-based protein–protein interaction analysis, multiple CytoHubba topology algorithms, transcriptomic evaluation, and exploratory machine-learning analysis. These downstream analyses were used to prioritize hypothesis-generating candidates and were not considered evidence of direct erythritol-induced gene regulation or molecular mediation.

### 4.4. Functional Enrichment Analysis

The overlapping genes underwent Gene Ontology (GO) and Kyoto Encyclopedia of Genes and Genomes (KEGG) enrichment analyses using the “clusterProfiler” R package (version 4.18.4), with a significance threshold of *p* < 0.05. Visualizations were generated using the “ggplot2”, “circlize”, and “ComplexHeatmap” R packages.

### 4.5. PPI Network Construction and Hub Gene Selection

Protein–protein interactions (PPIs) among the overlapping genes were examined using the Search Tool for the Retrieval of Interacting Genes/Proteins (STRING) database (https://string-db.org) with a medium confidence score threshold of >0.400. Isolated nodes were excluded to retain biologically relevant interactions. The resulting PPI network was visualized with Cytoscape 3.10.3 (https://cytoscape.org). To strictly identify the core hub genes, the CytoHubba plugin was employed. Eight distinct topological algorithms (MCC, DMNC, MNC, Degree, EPC, Bottleneck, EcCentricity, and Closeness) were applied, and the intersection of the top 30 ranked genes from each algorithm was established as the final set of hub genes.

### 4.6. Acquisition and Preprocessing of GEO Data

Two datasets from the GEO database were used for analysis. The GSE16561 dataset (platform: GPL6883), which includes 24 control and 39 IS peripheral blood samples, was designated as the training set. An additional dataset (GSE58294) was utilized as the independent validation set. Rather than performing global differential expression analysis, the expression matrices were directly preprocessed. Subsequently, the expression profiles of the initial 14 hub genes were extracted to construct the feature space for downstream machine learning.

### 4.7. Machine-Learning Prioritization of Candidate Genes

Three distinct machine learning algorithms were utilized to identify the most robust predictive features among the 14 candidate genes. First, LASSO regression was performed using the “glmnet” R package. A 10-fold cross-validation was applied to select the optimal penalty parameter. This step minimized binomial deviance and filtered out redundant variables. Second, the Support Vector Machine-Recursive Feature Elimination (SVM-RFE) algorithm was deployed via the “caret” and “e1071” packages. Incorporating a 10-fold cross-validation, it iteratively eliminated features with the lowest weights. This process successfully isolated highly discriminative genes. Third, a Random Forest (RF) model with 500 decision trees was constructed using the “randomForest” package. Feature importance was ranked by the Mean Decrease in Gini index. Strictly, the top 50% of these candidate genes were retained. Finally, the intersection of genes identified by all three algorithms was determined. The overlapping results were visualized using a Venn diagram and were used for exploratory candidate-gene prioritization. Model robustness was further evaluated using the leakage-resistant nested cross-validation analysis described below.

### 4.8. Leakage-Resistant Nested Cross-Validation Analysis

To further assess model robustness while minimizing potential information leakage, a repeated nested cross-validation analysis was performed using the 14 prespecified candidate genes and LASSO-penalized logistic regression. A stratified 5-fold outer cross-validation was repeated 10 times, with 5-fold inner cross-validation used for penalty selection according to the lambda.1se criterion. All preprocessing, feature selection, hyperparameter tuning, and model fitting were performed exclusively within each outer training fold, and the resulting model was applied unchanged to the corresponding held-out samples.

After nested cross-validation, a final LASSO model was fitted using the complete GSE16561 cohort and locked before external validation. The selected features, preprocessing parameters, penalty parameter, and model coefficients were applied unchanged to GSE58294, with no external data used for model optimization. This analysis served as an additional leakage-resistant robustness assessment of the original candidate-gene prioritization workflow.

### 4.9. SHAP Analysis

In this study, eight supervised machine-learning algorithms were used to classify stroke and control samples based on the identified candidate genes: Partial Least Squares (PLS), Logistic Regression (LR), glmBoost, Random Forest (RF), Decision Tree Structure (DTS), Support Vector Machine (SVM), Neural Network (Neural Net), and K-Nearest Neighbor (KNN). Model training and internal validation were rigorously executed using the “caret” R package with a 5-fold cross-validation strategy. Model performance was compared using Area Under the Curve (AUC), and the best-performing classifier in this exploratory comparison was selected for subsequent SHAP-based interpretation. To evaluate the best-performing model and elucidate its decision-making process, SHAP (SHapley Additive exPlanations) analysis was utilized. Based on cooperative game theory, this interpretable approach was implemented using the “kernelshap” and “shapviz” R packages. SHAP values were computed to quantify each feature’s specific contribution and relative importance to the predictive outcomes.

### 4.10. Construction and Evaluation of a Binary Logistic Regression Model

A binary logistic regression (LR) model was constructed based on the three prioritized stroke-related candidate genes (MMP9, TLR4, and HIF1A) to calculate the classification score for each sample. To improve model interpretability, a nomogram based on the three-gene classification model was generated using the rms R package. The predictive performance of the LR model was evaluated using receiver operating characteristic curve analysis, calibration curve analysis, and decision curve analysis. The independent dataset GSE58294 was used as an external validation cohort to assess the robustness and generalizability of the model.

### 4.11. Gene Set Variation Analysis (GSVA)

GSVA was performed using the GSVA R package to evaluate pathway-level differences associated with MMP9, TLR4, and HIF1A. Based on the median expression level of each gene, samples in GSE16561 were divided into high- and low-expression groups. Differentially enriched pathways were identified using limma, with *p* < 0.05 considered statistically significant.

### 4.12. Immune Cell Infiltration Analysis

Immune cell infiltration was evaluated using CIBERSORT in the stroke-related transcriptomic microenvironment. The LM22 reference matrix, which includes 547 marker genes and 22 human immune cell types, was used for immune deconvolution. Based on linear support vector regression, CIBERSORT was applied to the GSE16561 expression dataset to estimate the relative proportion of each immune cell subset. Group differences in immune cell infiltration were then assessed. Spearman correlation analysis was further performed to examine the associations between three prioritized stroke-related candidate genes, including MMP9, TLR4, and HIF1A, and immune cell infiltration levels.

### 4.13. Molecular Docking

Molecular docking was performed to explore the potential interactions between erythritol and the three prioritized candidate proteins. Three-dimensional protein structures were obtained from the RCSB Protein Data Bank (https://www.rcsb.org, accessed on 15 March 2026), and the molecular structure of erythritol was retrieved from PubChem (https://pubchem.ncbi.nlm.nih.gov, accessed on 15 March 2026). Considering the small molecular size and high polarity of erythritol, docking analysis was used as an exploratory approach to evaluate possible ligand–receptor binding. Docking was conducted using the CB-Dock2 online platform (https://cadd.labshare.cn/cb-dock2, accessed on 15 March 2026) [43]. Binding affinity was assessed according to the predicted binding energy, with values below 0 kcal/mol indicating potential spontaneous binding and values below −4 kcal/mol considered to suggest relatively stable binding in this study.

## 5. Conclusions

In conclusion, this study provides genetic evidence linking higher genetically predicted circulating erythritol levels with stroke risk. These findings concern circulating erythritol and do not directly establish that dietary erythritol intake causes stroke, because circulating levels may also reflect endogenous production, metabolism, and renal handling. Nevertheless, because dietary erythritol can increase circulating concentrations, the observed association raises a potential safety signal and supports prospective and interventional evaluation of high or sustained erythritol consumption, particularly in individuals at elevated cardiovascular and cerebrovascular risk.

## Data Availability

The data that support the findings of this study are available in the Gene Expression Omnibus (GEO) at https://www.ncbi.nlm.nih.gov/geo/ (accessed on 10 March 2026), reference numbers GSE16561 and GSE58294. These data were derived from the following resources available in the public domain: GSE16561, https://www.ncbi.nlm.nih.gov/geo/query/acc.cgi?acc=GSE16561 (accessed on 10 March 2026); GSE58294, https://www.ncbi.nlm.nih.gov/geo/query/acc.cgi?acc=GSE58294 (accessed on 10 March 2026).

## Supplementary Materials

The following supporting information can be downloaded at: https://www.mdpi.com/article/10.3390/ijms27177811/s1.
