# Supplementary material for: Risk of Erythritol-Associated Ischemic Stroke: Integrated Genetic, Transcriptomic, and Machine Learning Evidence
Source: Int J Mol Sci. 2026 Aug 31;27(17):7811. doi: 10.3390/ijms27177811 (PMC13566553; doi:10.3390/ijms27177811)

Supplementary Figures

Supplementary Figure S1. Sensitivity analysis for the MR association between erythritol and ischemic stroke

(A) Scatter plot

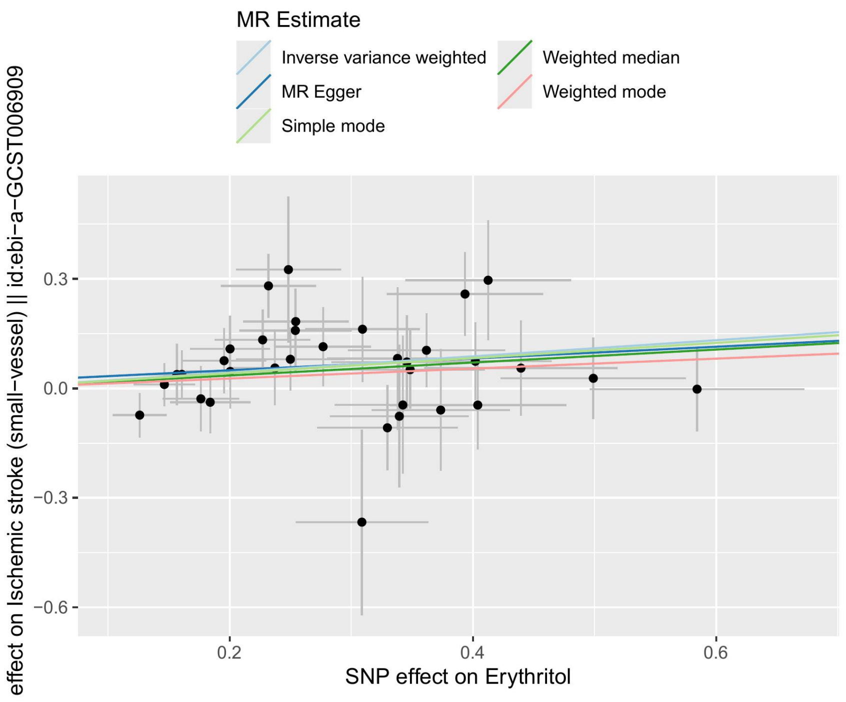

(B) Funnel plot

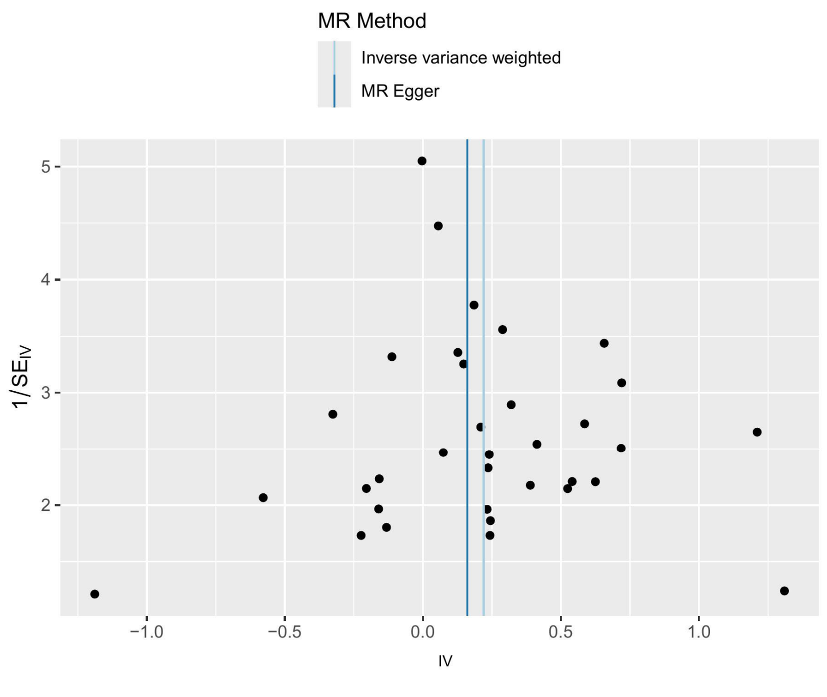

(C) Leave-one-out analysis

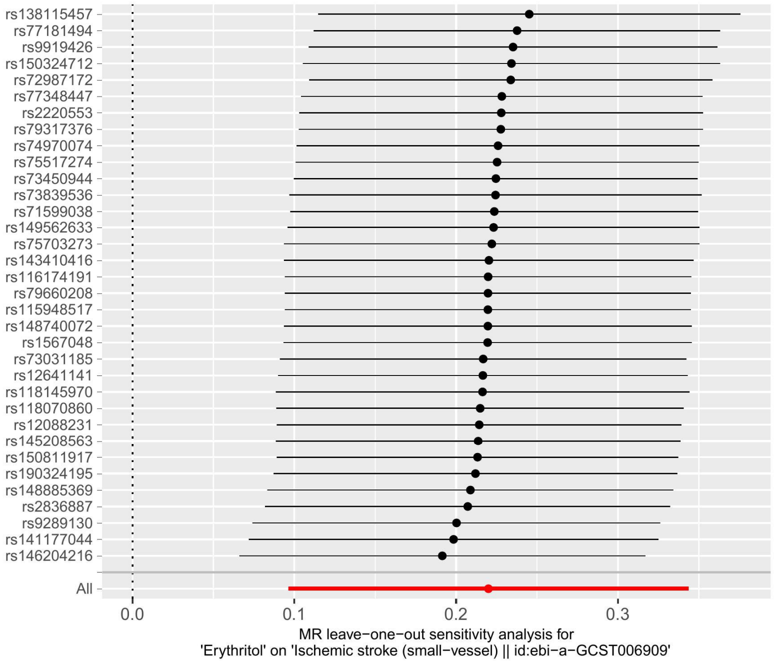

Supplementary Figure S2. Leakage-resistant nested LASSO analysis and external validation.
(A) Pooled out-of-fold ROC curve from repeated nested LASSO cross-validation in GSE16561 (AUC = 0.960, 95% CI: 0.912–1.000).

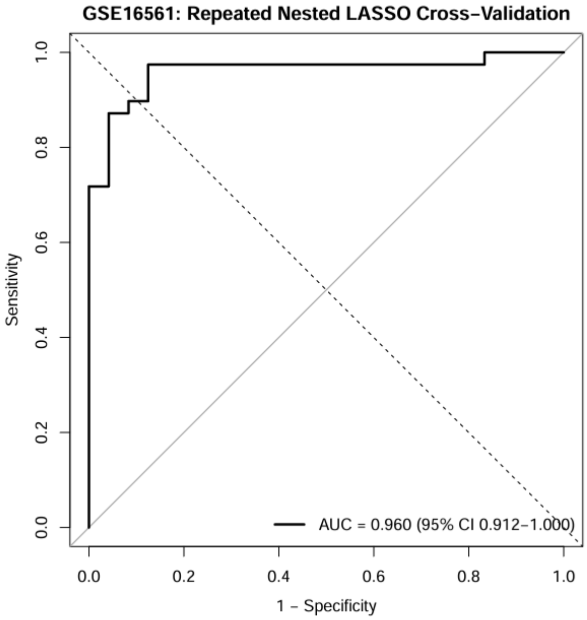

(B) Selection frequencies of the 14 prespecified candidate genes across outer training folds.

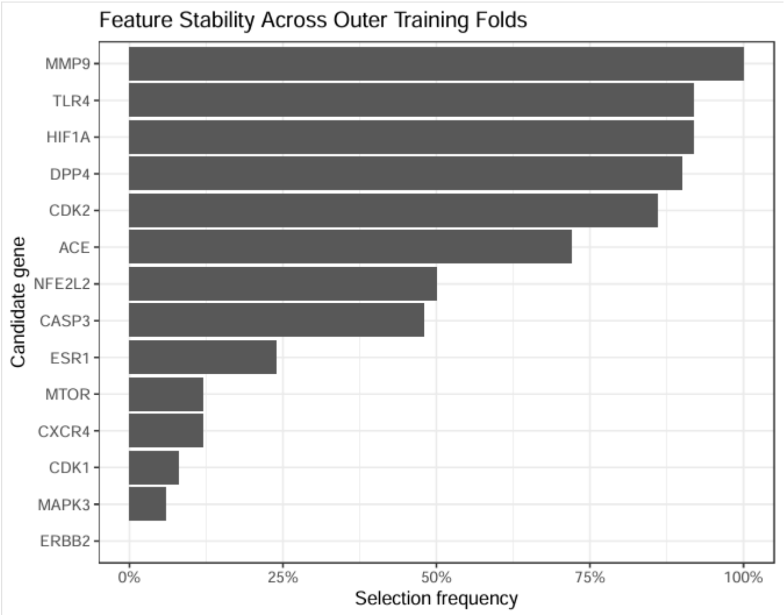

(C) ROC curve of the locked final LASSO model in the independent GSE58294 cohort (AUC = 0.802, 95% CI: 0.711–0.892).

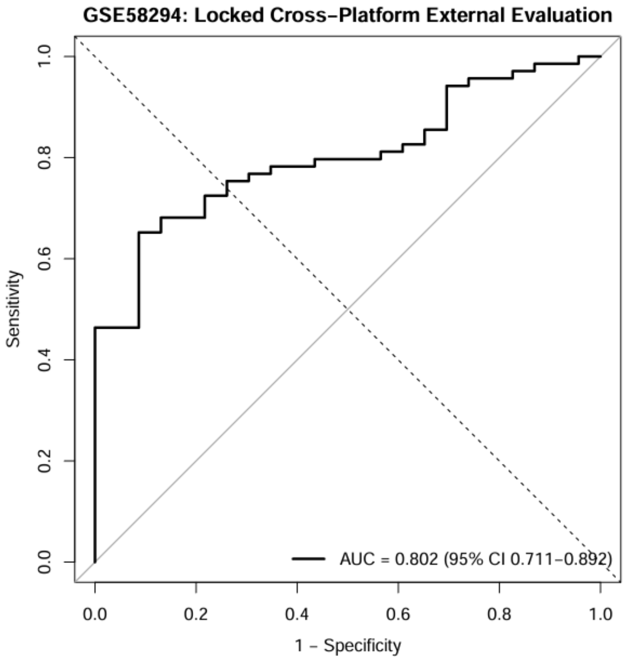

Supplement: Supplementary file 1 [file ijms-27-07811-s001.zip › Supplementary_Figures_MR_Artificial_Sweeteners.pdf]
